# A ultra-stable point of care nanozyme-based kit for cTnI detection in human serum

**DOI:** 10.3389/fbioe.2025.1570668

**Published:** 2025-05-15

**Authors:** Mingfei Sun, Zhan Yang, Ziwei Ma, Jinbo Wu, Shaoying Lu

**Affiliations:** ^1^ Department of Vascular Surgery, The First Affiliated Hospital of Xi’an JiaoTong University, Xi’an, Shaanxi, China; ^2^ Department of Vascular Surgery, The First Affiliated Hospital of Henan University, Kaifeng, Henan, China; ^3^ Joint National Laboratory for Antibody Drug Engineering, Henan University, Kaifeng, Henan, China

**Keywords:** cTnI, iron oxide superparamagnetic nanoparticles, nanozyme, lateral flow immunoassay, acute myocardial infarction

## Abstract

Home discovery of myocardial infarction can significantly improve the rate of treatment. Cardiac troponin I (cTnI), as a biochemical marker that has been introduced into clinical diagnosis and treatment guidelines, can effectively predict the occurrence of myocardial infarction. We constructed highly stable nanozyme based on Fe_3_O_4_ nanoparticles and prepared rapid detection reagents for myocardial infarction by modifying anti-cTnI antibodies. The results showed that the nanozyme with an average particle size of 200 nm had peroxidase activity and could effectively catalyze 3,3′, 5,5′-tetramethylbenzidine (TMB). With a sensitivity of up to 1.5 ng/mL, the kit showed superior performance than commercial colloidal gold assay kits, and could effectively detect cTnI in serum with an overall compliance rate of 92.1%. The study provides a new approach to home detection of heart attacks.

## Introduction

In recent years, there has been a significant increase in the number of deaths attributed to cardiovascular disease, with a notable trend towards younger individuals being affected (Guo et al., 2018). Approximately 20 million people succumb to cardiovascular diseases annually, accounting for one-third of all global fatalities. Among these conditions, acute myocardial infarction (AMI) stands out as the most critical risk factor for cardiovascular disease (Wu et al., 2020). AMI is a medical emergency characterized by coronary artery occlusion leading to myocardial ischemia and necrosis (Pandey et al., 2011). Patients typically present symptoms such as chest pain, dyspnea, and even shock-induced death. The 3-h window following onset is crucial for timely intervention, as prompt diagnosis and treatment can maximize restoration of myocardial function and significantly enhance patient survival rates (Guo et al., 2023). Therefore, early diagnosis plays an important role in the prevention and treatment of AMI. Days or weeks before the onset of myocardial infarction, most patients will experience prodromal symptoms, Typical symptoms include chest pain or discomfort behind the sternum, shortness of breath, arm or shoulder pain, weakness, dizziness or even fainting; atypical symptoms include stomach or abdominal pain, nausea and vomiting, headache, and facial, mouth or dental pain and some of the underlying symptoms are undetectable (Birnbach et al., 2020). Regular checkups can detect most of the risk factors, reduce the incidence of myocardial infarction, and increase the cure rate, especially for people with a high prevalence of myocardial infarction, such as those with a family history of genetic predisposition, a history of healing, and related cardiovascular system diseases. However, most patients do not pay enough attention to this, and early clinical diagnosis requires a lot of time and effort, which reduces patient compliance, thus delaying the diagnosis and treatment of myocardial infarction (Reed et al., 2017). Consequently, there is an urgent need for a convenient yet feasible home-based detection method that enables early recognition of myocardial infarctions so that patients can promptly seek appropriate medical attention.

Point-of-care testing (POCT) technology is developing rapidly, among which Lateral Flow Immunoassay (LFIA) is widely used for home self-examination and bedside rapid testing because of its rapid and low-cost, simple and easy-to-read characteristics (Liu S. et al., 2024). The labeling probe is one of the core components of LFIA, which largely affects the accuracy and sensitivity of the assay (Sena-Torralba et al., 2022). With the development of clinical testing technology and the in-depth study of diseases, the requirement of detection refinement becomes more and more important, and the immediate detection technology also faces more challenges. Traditional colloidal gold probes have been used in a variety of disease detection with a mature preparation process, which has brought great convenience to clinical diagnosis. However, due to the low molar extinction coefficient, in some aspects of the detection can not meet the sensitivity requirements, the degree of color rendering of the probe is closely related to the results of the naked eye observation, the low sensitivity will have a great risk of misjudgment, to the family self-test and clinical diagnosis brings unnecessary trouble (Dong et al., 2024), as a result many novel probes have emerged, such as: nanozymes (Liang et al., 2023), carbon nanoparticles (Ansari et al., 2024), silica nanoparticles (Liang et al., 2024), SeNPs (Chen et al., 2024), PtNPs (Kim et al., 2017), etc. However, carbon nanoparticle preparation is challenging and expensive while silica nanoparticles lack a colorimetric effect and often require combination with other materials. Nanozyme are a class of nanomaterials with enzyme catalytic activity that are also paramagnetic, allowing stable and controlled catalytic (Liu et al., 2011). Numerous studies have employed nanozymes as probes for cancer and virus detection which have demonstrated advantages including high sensitivity, low cost, stability among others. Wang et al., for instance used Fe_3_O_4_ nanozymes for sensitive extracellular vesicle detection (Wang B. et al., 2022). Liu et al., employed Fe_3_O_4_ nanoparticles for agricultural residue detection (Liu S. et al., 2024). We envision utilizing nanozymes in early diagnosis of AMI where myoglobin (Myo), cardiac troponin (cTn), and creatine kinase- MB (CK-MB) currently serve as three key indicators in clinical practice (Guo et al., 2023). Among these markers Myo exhibits poor specificity while CK-MB demonstrates low sensitivity, cTnI and cTnT are sensitive for the characterization of myocardial injury (Jaffe, 2006). However, in some cases, such as skeletal muscle injury, elevated cTnT has also been reported (Yildirim et al., 2024). Cardiac troponin I has both high specificity and high sensitivity and is the golden standard for the diagnosis of AMI (Yildirim et al., 2024).

In this study, to improve the sensitivity, nanoparticle enzymes (ENPs) were selected as probes to construct the FLIA platform, which is a method to transfer the traditional ELISA to LFIA, with strong anti-interference ability and more objective and accurate results. We used hydrothermal method to synthesize homogeneous and stable nanoparticles of the enzyme with a particle size of about 200 nm (Higgins and Higgins, 2003) and characterized through dynamic light scattering (DLS), transmission electron microscopy (TEM), scanning electron microscopy (SEM), etc. The nanozymes were employed as labeled probes to successfully develop a point-of-care ENPs-based LFIA (E-LFIA) for cTnI. The LFIA exhibits excellent sensitivity and stability, surpassing that of commercial colloidal AuNPs-LFIA (Au-LFIA). Seventy-six clinical human plasma samples were tested, resulting in an overall coincidence rate of 92.1% compared to ELISA. The comprehensive results show that the E-LFIA has highly credible analytical performance and broad application prospects.

## Materials and methods

### Preparation and morphology analysis of ENPs

The nanoparticle enzymes (ENPs) were synthesized using the hydrothermal method as described in the literature (Liang et al., 2013). A total of 0.6 g of ferric chloride crystal (FeCl_3_·6H_2_O) and 1.5 g of sodium acetate were sequentially added to 20 mL of ethylene glycol. After vigorous stirring for 30 min, the mixture was heated at a temperature of 200°C for a duration of 16 h, followed by washing with alcohol and drying at a temperature of 60°C. Morphology and dispersion were observed with the help of TEM and SEM and particle size and potential were analyzed using DLS.

### Anti-cTnI antibody was conjugated to the ENPs

The anti-cTnI antibody was coupled with the ENPs according to literature reports (Atta et al., 2024). 5 mg of EDC and NHS were dissolved in 1 mL of ultrapure water, 5 mg of ENPs were added, incubated for 30 min at room temperature and then collected magnetically. It were incubated overnight at 4°C after washing with ultrapure water, and the nanoparticles were collected using the magnet and washed with PBS and then incubated for 30 min in Tris-HCl buffer (50 mM, pH 7.2) and preserved in a 5% BSA-containing PBS solution for backup.

### Assembly of the cTnI E-LFIA and interpretation of the results

**FIGURE 1:**
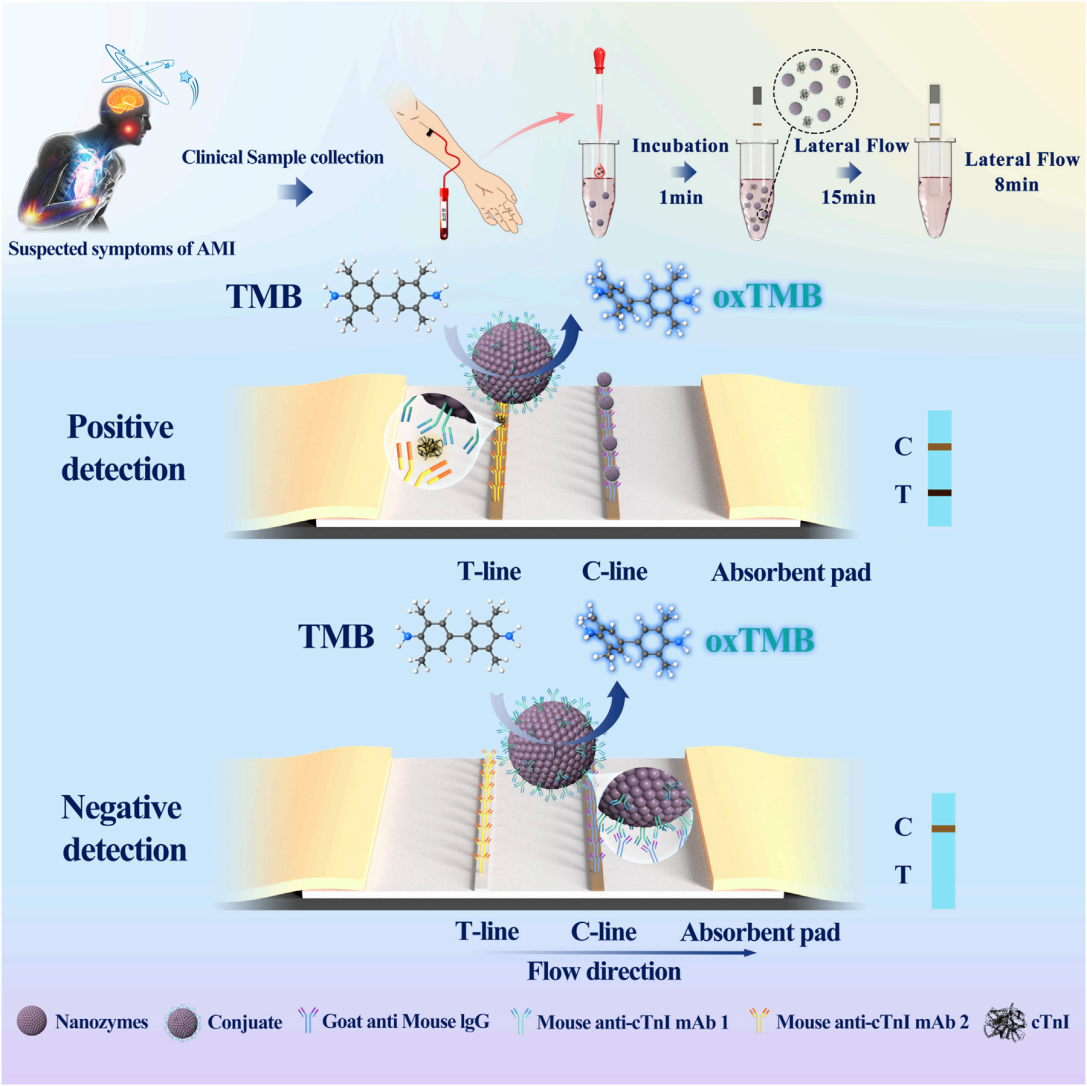
Schemas of cTnI E-LFIA. (Nanozymes: ENPs; Mouse anti-cTnI 1: a monoclonal antibody to the cTnI; Mouse anti-cTnI 2: another monoclonal antibody to cTnI, distinguished from Mouse anti-cTnI 1).

E-LFIA preparation and assembly were performed according to the previous studies of our group (Chen et al., 2021). 1 mg/mL cTnI monoclonal antibody (distinguished from the labeled antibodies described above) and goat anti-mouse IgG were used as the test line (T Line) and quality control line (C Line), respectively, and the T line and C line were drawn on the nitrocellulose (NC) membrane by using a membrane scribe, which was the reaction pad. After drying at 37°C for 1 h, enables antibodies to be thoroughly piggybacked on the NC membrane, the reaction pad was incubated in 1% BSA solution for 30 min at room temperature and washed with borate buffer for 3 times to close the optimization of the chromatographic background, and then it was dried at 37°C for 3 h again and then pasted onto a base plate, cutting and assembly. The detection principle and process are as follows (Figure 1), cTnI magnetic particles were stored in the tube, gently mixed with the sample solution for 1 min, and then the test strip was placed in the tube at a downward inclination, with the reaction pad extending about 5 mm below the liquid surface, not allowing the liquid surface to touch the T line, and the sample solution mixed with the nanozyme particles was chromatographed upward along the binding pad under capillary action, until it reached the upper absorbent pad. The whole process lasted 15 min to achieve the optimal chromatographic effect. Then the chromatography test paper was inserted into the tube of color developing liquid in the same way. The color developing liquid contained H_2_O_2_ and the color developing substrate TMB. The chromatography test paper was allowed to develop chromatographic color again for 8 min for result observation. When the C line showed color, if the T line showed color, it was positive; if not, it was negative. If the C line did not show color, the results were not credible regardless of the state of the T line.

### Detection sensitivity is an important indicator of the performance of an assay method

With the progression of diseases and the deepening of people’s understanding of diseases, the demands for reducing the detection limit of biomarkers are constantly growing, and the detection of substances at extremely low concentrations is an urgent challenge that needs to be addressed. Immediate detection has the advantage of rapid and convenient operation. For lateral immunochromatography, the lower the detection limit, the more sensitive the detection of the target is, and the more it reflects the high performance of the LFIA (Liu et al., 2023). Therefore, we used the antibody dilution solution to dilute the cTnI standard to 500, 10, 5, 2, 1.5, 1 ng/mL, and inserted the E-LFIA into different concentrations of cTnI standard solutions. After allowing them to react for 15 min, TMB color development was carried out for 8 min to observe the lowest color-developing concentration, which was regarded as the lowest detection limit.

### Superior specificity is necessary for detection of target biomarkers

The target of the E-LFIA is cTnI, and the ultimate goal is to perform a rapid diagnosis of myocardial infarction, in which there will be abnormal levels of multiple biomarkers, and we need to exclude these interferences in order to show the specificity and high accuracy of our test target. For this reason, we chose some biomarkers related to myocardial injury, such as H-FABP (Wang L. et al., 2022), CK-MB, and Myo, as interference items, and the concentration of each marker was set to 5 μg/mL, and under the influence of such a high concentration of interferences, the assay was performed and the color development was observed, respectively, to validate the specificity of the test strips for the detection of cTnI.The stability of the LFIA is also an important goal to examine. Different batches of prepared LFIA must maintain the same detection performance, and finally present the same detection results. For this reason, we prepared three batches of LFIA at different times and verified the detection limits of each batch of LFIA according to the sensitivity test method to prove the stability of the preparation method and technology.

### High stability leads to stable and reliable results and is an indication of the maturity of the preparation process and experimental methodology

The stability of the LFIA is also an important objective to be examined; random results cannot be a valuable reference while reproducible results are required. Stability includes differences between batches and time - dependent studies (or accelerated failure). For different batches of LFIA preparation, it is important to maintain the same detection performance, and finally they present the same test results. If different batches show different performance, it means that the preparation process still needs to be optimized or there are deviations in the preparation process, so the results obviously do not have reference value, and may even be misleading for patients and clinical staff. For this reason, we prepared three batches of LFIA at different times under optimal conditions and verified the detection limits of each batch according to the sensitivity test method to prove the stability of the preparation method and technology. For the time stability experiment, we adopted a similar approach, sealed the prepared test strip and stored it in a light-free environment at 40°C and humidity below 30%, and took it out for detection on the fifth, 10th and 15th days to verify its detection limit.

### Real clinical samples are tested to verify the actual effectiveness of the E-LFIA

The cTnI E − LFIA is intended for use in clinical testing. Clinical samples are more difficult to test than dilutions of standards. The contents of clinical serum samples are complex, and the patient’s disease status has a significant impact on blood biochemical indexes. In order to judge the actual effectiveness of the LFIA, we must use real clinical samples to observe the chromatographic effect and color development. The selection of clinical samples should be representative. We selected 36 human fresh plasma samples from high, middle and low different age groups, with an equal proportion of men and women, to conduct a double - blind experiment using ELISA and our homemade E − LFIA for detection respectively. With a cTnI concentration of 1.5 ng/mL as the critical value, if the concentration is higher than 1.5 ng/mL, it is considered positive, and *vice versa* for negative, to validate the effect of the LFIA and analyze the correlation between the results. Analyzing the correlation of the results.

Clinical samples were collected. They were confirmed by clinical detection at the First Affiliated Hospital of Henan University. The sample analysis of patients was conducted at the First Affiliated Hospital of Henan University by clinical staff who followed the test procedure described in the specification. This study was approved by the Biomedical Research Ethics Subcommittee of Henan University (HUSOM - 2022 - 052). All the experiments were performed in compliance with the relevant laws and institutional guidelines, and the clinical sample contributors knew and agreed to this research.

### Comparison with commercial colloidal Au-NPs to present advantages and disadvantages

Commercially available cTnI colloidal Au-LFIA have been commonly used. We wished to judge the performance of the homemade E-LFIA in this study by comparing it with the commercially available colloidal Au-LFIA. The important item for comparison of the performance is the lowest detection concentration. For this reason, we prepared different concentrations of cTnI standard solutions and tested them with the above two LFIA. By observing the color development effect and comparing the lowest color development concentration, in this way, we can judge the detection sensitivity of the two methods and compare the performance of the LFIA.

### Image acquisition and data processing

Images are captured by a Canon digital camera to ensure bright light and clear images for analysis needs. The grayscale analysis was performed by ImageJ software, and the statistical graphs were drawn by Origin software. Each set of experiments was repeated at least 3 times to ensure stable and reliable results.

### Supplementary material

The sources of reagents required for the study are documented in the additional material. Original images and patient clinics are also presented in the additional material.

## Results

### Chromogenic enhancement principle and preparative characterization of ENPs

**FIGURE 2:**
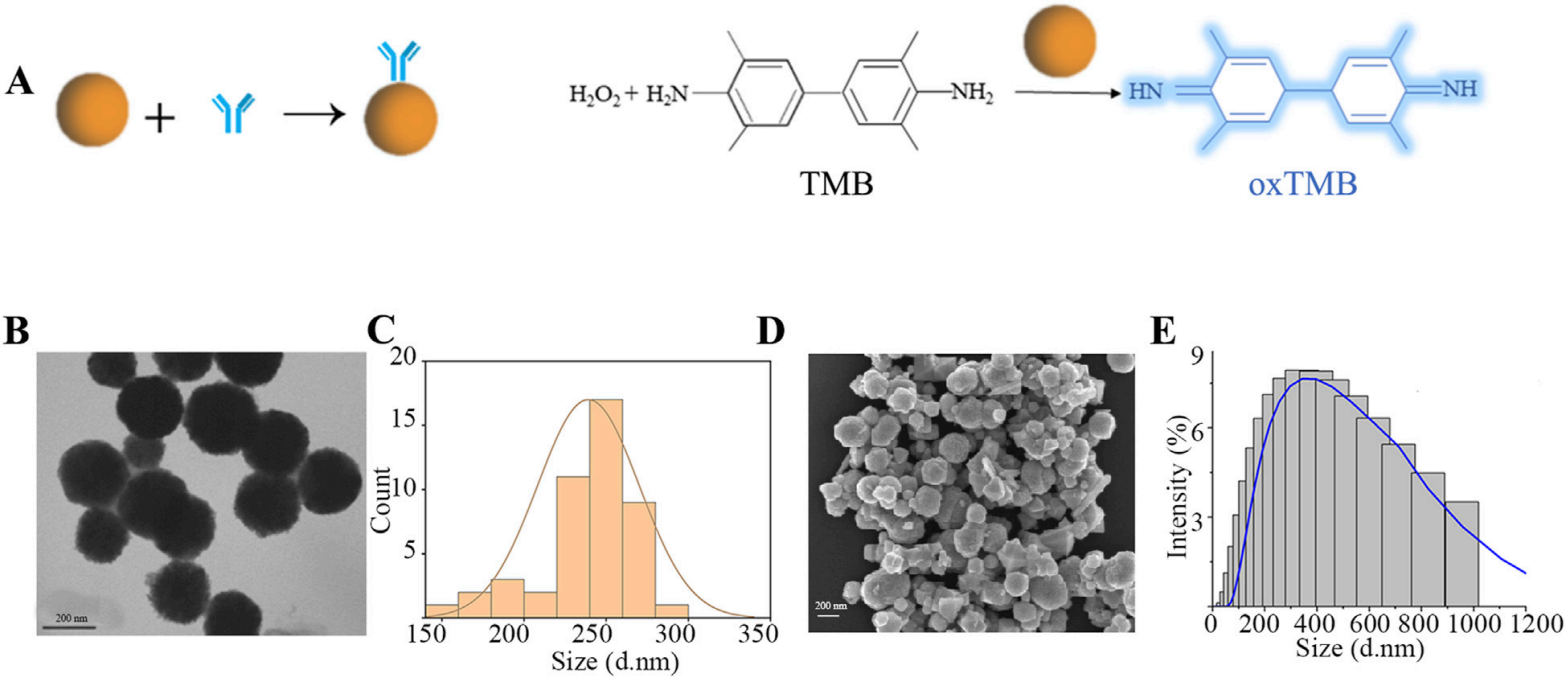
Preparation and characterization of ENPs. **(A)** ENPs coupled with mouse anti-cTnI one and catalytic principles; **(B)** TEM characterization; **(C)** Dry particle size distribution; **(D)** SEM characterization; **(E)** Hydrated particle size distribution.

The nanoparticle enzymes (ENPs) derive their name from their peroxidase activity, a property that allows them to catalyze the colorless TMB to blue oxidized TMB in the presence of hydrogen peroxide (Figure 2A). This is also the principle of signal amplification that we utilize in secondary chromatography. In this paper, the preparation of ENPs was carried out in the liquid phase using FeCl_3_-6H_2_O and NaAc as reaction substrates. In order to test the properties of these ENPs, the morphology was observed using TEM and SEM (Figures 2B–D); meanwhile, the particle size of the nanoparticles of the nanozymes was analyzed by DLS. It was verified that the prepared nanoparticles had a dry particle size of about 200 nm, a uniformly distributed spherical shape, and a hydrodynamic particle size of about 400 nm, and they were well dispersed in the aqueous phase system with excellent chromatographic effect (Figure 2E). According to the commonly used method reported in the literature, the mouse anti-cTnI one was coupled with the ENPs, which involves activation with NHS and EDC, so that the antibody binds to the carboxyl group on the nanozymes particles to form an amide bond, which is ultimately used for the preparation of LFIA.

### The lowest observed concentration of the kit by visual colorimetry was 1.5 ng/mL

**FIGURE 3:**
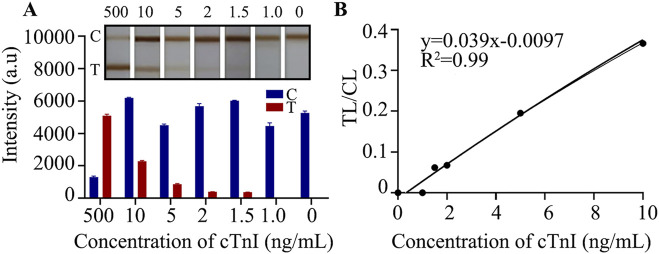
Detection limit analysis. **(A)** The effect of gradient sample detection; **(B)** Linear analysis.

cTnI is present in human serum at very low concentrations, usually at the picogram per milliliter to nanogram per milliliter level. Improving the sensitivity of the assay allows us to obtain more accurate changes in the levels of the target assay, which can accurately reflect the disease state, which is what we have been pursuing. We diluted the cTnI standard to 500, 10, 5, 2, 1.5, and 1 ng/mL under optimal preparation conditions and used these dilutions for the following assays. LFIA were inserted into different concentrations of cTnI standard solutions and allowed to react for 15 min. Subsequently, TMB color - development was carried out for 8 min to determine the detection limit. The results showed (Figure 3A) that the chromatography test paper T line could still show color when the concentration of cTnI was 1.5 ng/mL, and could not show color when it was 1.0 ng/mL, which indicated that the detection limit of the E − LFIA was 1.5 ng/mL. The original image is shown in Supplementary Figure S1. After we took pictures using a smartphone or camera, we analyzed the gray values with the help of ImageJ software and calculated that the ratio of the color - rendering intensity of the T - line to the C - line had a good linear correlation with the cTnI concentration with an R2 value of 0.99 within the concentration range of 1.5–10 ng/mL (Figure 3B). Accordingly, we can realize the quantitative analysis of cTnI in the assay based on the value of TL/CL and the standard curve in the linear range. In the detection using immunochromatographic test strips, factors such as different batches of test strips, sample loading amounts, and detection environments may affect the signal intensity of the T line. By establishing the TL/CL ratio method, the influence of some interfering factors can be eliminated (Xu et al., 2023). The T/C value can, to a certain extent, reflect the degree of the immune response, enhance the sensitivity and accuracy of the detection, establish a better functional relationship, and contribute to the promotion of quantitative detection (Cheng et al., 2014).

### The E-LFIA showed excellent specificity in the presence of different interferents

**FIGURE 4:**
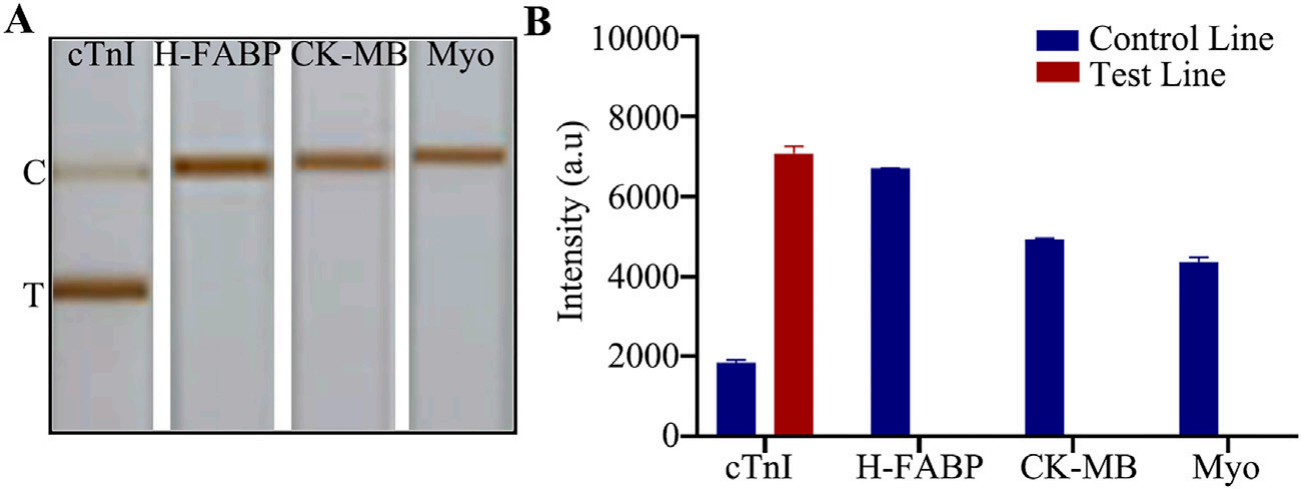
Specific detection. **(A)** Detection of myocardial infarction markers; **(B)** Quantitative analysis.

A high degree of specificity is necessary for the detection of a particular biomarker, especially for complex samples. Chromatography test strips are very popular among users for their quick and easy reading, we only need to observe whether the T-line shows color or not to judge the result. In addition to the test target, we need to exclude any other substances that may affect the color change of the T-line. For cTnI, our goal is to only observe a gray change in the T-line when detecting cTnI, while any other substances should not cause color buildup in the T-line. cTnI, as a gold indicator of myocardial infarction, shows significant changes in levels during myocardial injury. Similar biomarkers of myocardial injury are H-FABP, CK-MB, and Myo. Therefore, we prepared high concentrations of the above components, all at 5 μg/mL. In the presence of this high concentration of interferences, the specificity and accuracy of the assay results can be reflected. After comparison, we found that the T - line of the chromatography LFIA showed color only when detecting cTnI, and the rest of the components did not show positivity (Figures 4A, B), which indicated that the test paper did not cross - react with other infarction markers and had excellent specificity. The original image is shown in Supplementary Figure S2.

### The E-LFIA have a high degree of stability and present trustworthy results

**FIGURE 5:**
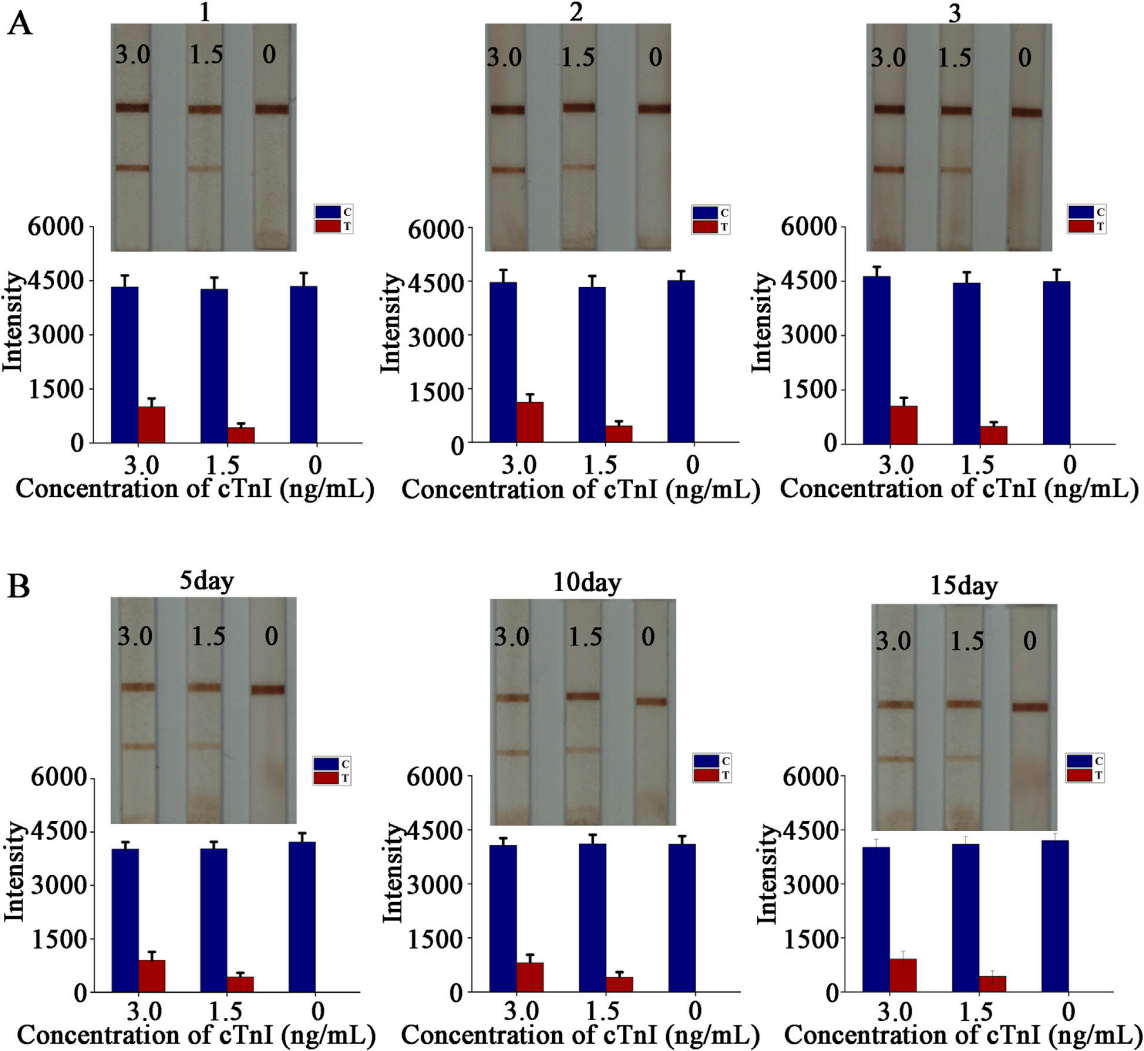
Stability experiment. **(A)** Stability test between batches and quantitative analysis; **(B)** Time-dependent study and quantitative analysis.

The reproduction of test results is an important way to measure the maturity and stability of a method or process. The LFIA is ultimately used in the clinic, and the results it presents are directly related to the health of the patient. Occasional or one - off results cannot be taken as a reliable assessment, and even the occurrence of erroneous results that mislead the patient or the healthcare worker is an outcome that none of us would like to see. We need to successfully repeat the same results in multiple experiments to determine that stable and reliable results are obtained, which also reflects the maturity of the design method and preparation process. A common method is to compare the variability of test results from different batches of LFIA. To this end, we prepared three batches of LFIA on different dates, and verified them according to the sensitivity analysis method described above. The test results showed that the lowest color - developed concentration of the three batches of LFIA reached 1.5 ng/mL (Figure 5A). The original image is shown in Supplementary Figure S3. Different batches of LFIA showed the same sensitivity, which proved that the batch - to - batch stability was strong.Meanwhile, we conducted a time - dependent study to explore its reliable storage time. After preparing the strip, we sealed it and stored it in a light - free environment at a temperature of 40°C and with humidity below 30%. We took it out for testing on the fifth, 10th and 15th days respectively. The results showed that the detection limit reached 1.5 ng/mL (Figure 5B), and the strip still showed a clear color, indicating good stability.

### Clinical sample test results show excellent compliance rates

**FIGURE 6:**
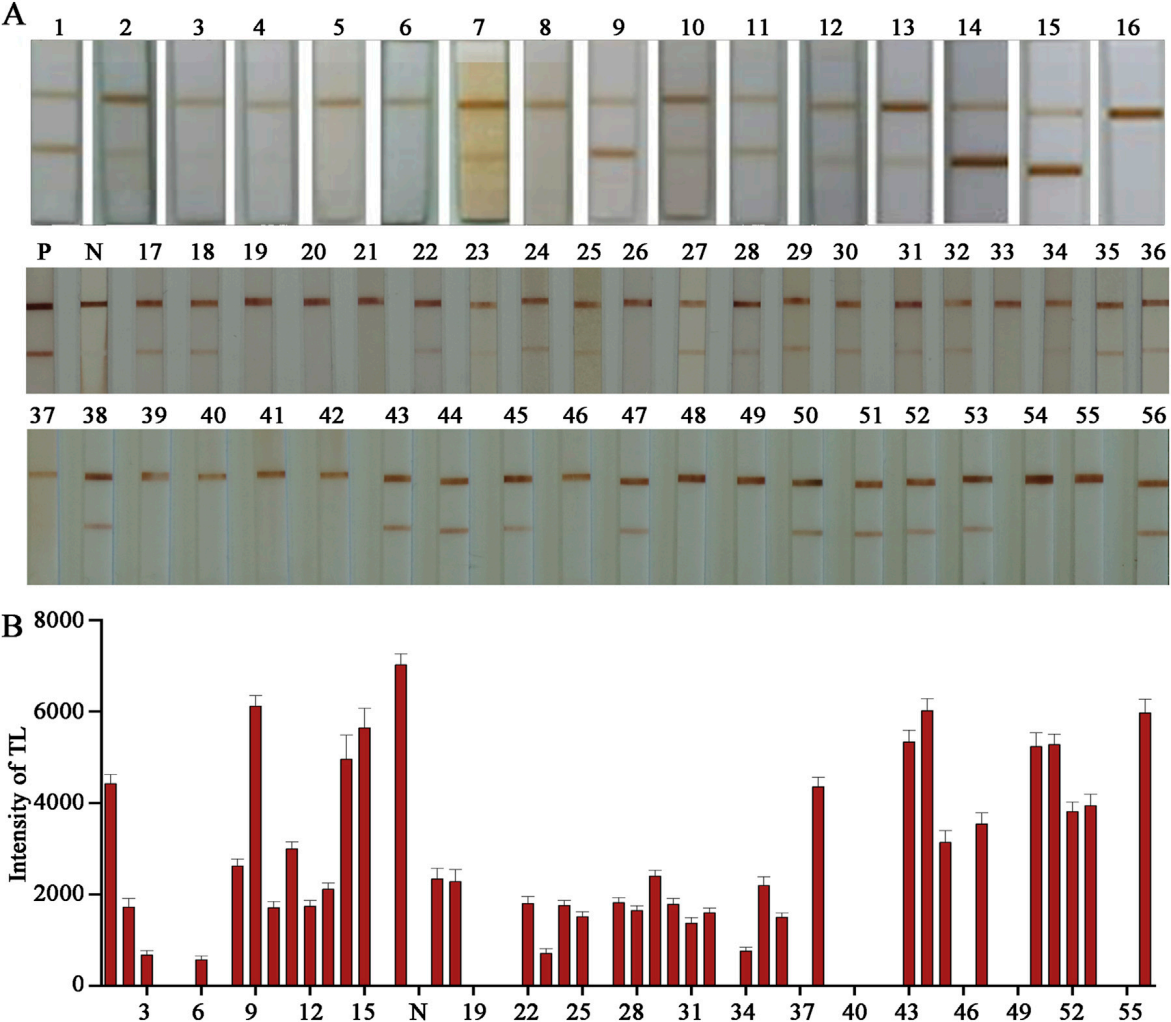
Detection of clinical samples. **(A)** Actual test picture; **(B)** Quantitative analysis.

Our ultimate expectation of developing cTnI E − LFIA is to serve clinical diagnosis or family self - test. The test sample is serum, which has a more complex composition compared to the standard solution and may have a greater impact on the entire chromatographic process and color - development results of the LFIA. To reflect the most realistic application, the test must be performed on real clinical plasma samples. When conducting the sample test, certain conditions are also required. The selected serum samples need to be random and representative. Finally, 36 cases of human fresh plasma clinical samples covering high, middle, and low three different age groups were selected, in which the proportion of men and women was equal. During the detection, double - blind experiments were carried out using ELISA and our homemade E − LFIA respectively. With 1.5 ng/mL as the threshold, if the concentration is higher than 1.5 ng/mL, it is positive; *vice versa*, it is negative. This was to validate the LFIA detection effect and analyze the correlation of the results. The testing process was double - blind, hiding the information of the test samples and eliminating as much as possible the interference of personal subjective will in the testing process. The final results of the ELISA test were 42 positive cases and 34 negative cases. The results of the E − LFIA were 36 positive cases and 36 negative cases, and only 6 cases of weak positivity were not detected (Figures 6A, B). Where “P” and “N” represent positive standard and negative standard respectively. After tracing, the index of six undetected positive samples was low, and the serum cholesterol content was high, which had a certain impact on the detection result. Compared with the ELISA results, the positive coincidence rate was 85.7%, the negative coincidence rate was 100%, and the overall coincidence rate was 92.1%. It shows good performance. The raw images of the test - strip results are shown in Supplementary Figure S4. The positive patient information from which the samples were derived for the anticipated analysis of the results is summarized in Supplementary Table S1, and the negative patient information is in Supplementary Table S2.

### The E-LFIA prepared in this study showed superior performance over commercially available Au-LFIA

**FIGURE 7:**
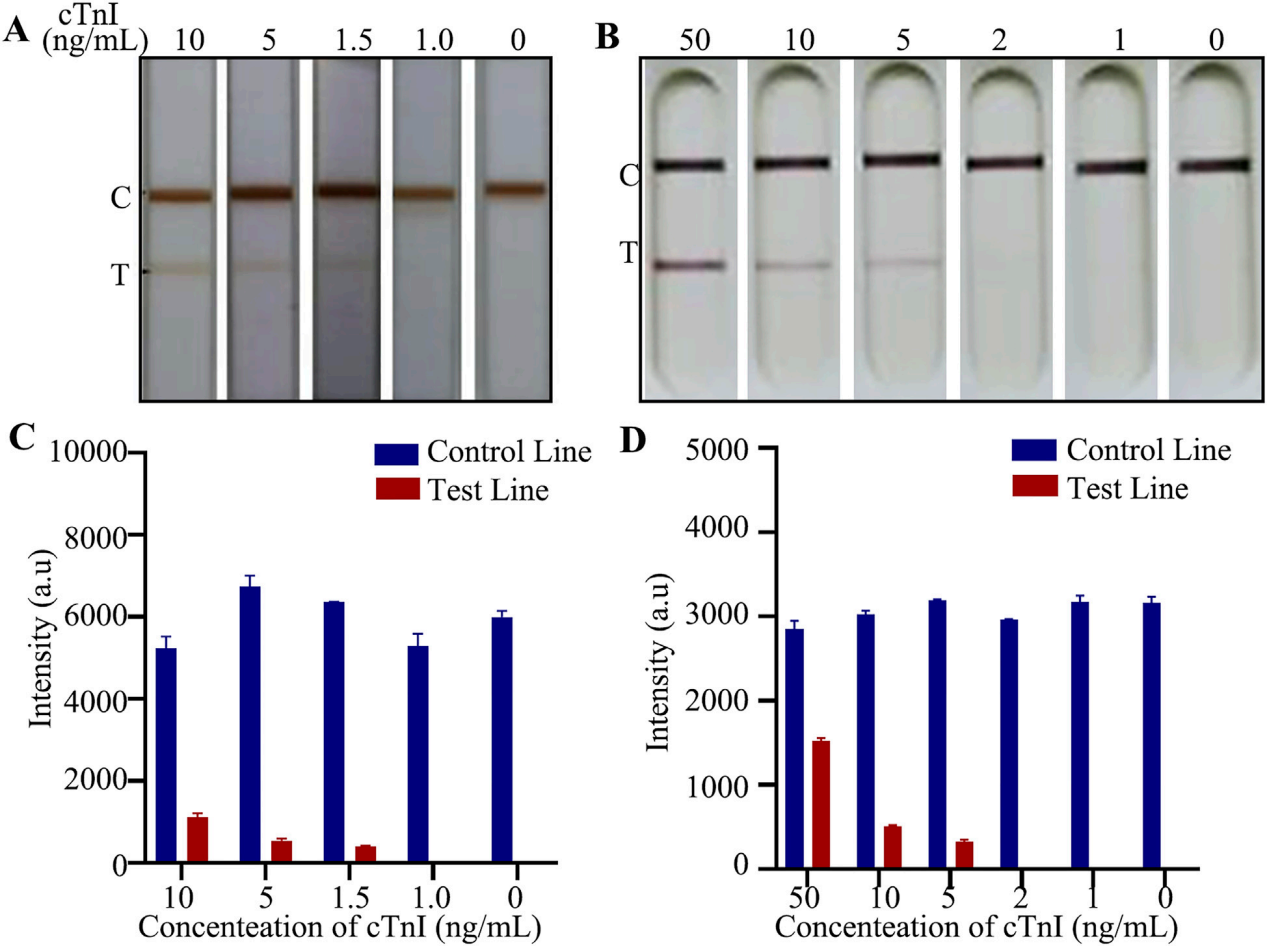
Comparison of detection effect of LFIA. **(A)** Actual test picture of self-made E-LFIA; **(B)** Actual test picture of commercial Au-LFIA; **(C)** Quantitative analysis of E-LFIA; **(D)** Quantitative analysis of Au-LFIA.

The most established lateral immunochromatographic method is the AuNPs, and commercialized Au-LFIA exist on the market and have been accepted by the general public. We considered the commercialized Au-LFIA as a reference, and compared the purchased Au-LFIA with the E-LFIA prepared in this study to measure the superiority of our assay. The most effective item for comparison is the lowest detectable concentration. For this purpose, we prepared different concentrations of cTnI standard solutions and detected them with the two LFIA mentioned above, respectively. Under the premise that the C-line is visible, the lowest color-developed concentration of the cTnI E-LFIA prepared in this study was 1.5 ng/mL; whereas the lowest color-developed concentration of the commercial cTnI Au-LFIA was 5 ng/mL, which is a difference of about 3.3-fold (Figures 7A, B). The original image is shown in Supplementary Figure S5. It can be seen that the sensitivity of the cTnI LFIA was significantly improved when ENPs were used as the labeling probe. cTnI exists in human blood at a very low concentration, and the lower detection limit can reflect the changes of the index more sensitively, which is more suitable for the detection of AMI.

## Discussion

With the development of social economy and the change of national lifestyle, especially the acceleration of population aging and urbanization, the unhealthy lifestyle of residents has become increasingly prominent, and the incidence of cardiovascular disease continues to increase. According to the World Health Organization (WHO), by 2030, more than 23.3 million people will die of cardiovascular disease each year (Zhang et al., 2022). At present, cardiovascular disease accounted for the first cause of death in both urban and rural residents in China, which was 46.66% in rural areas and 43.81% in urban areas (Liu M. et al., 2024). The economic burden of cardiovascular disease on residents and society is increasing. It is estimated that there are 330 million people suffering from cardiovascular disease in China, accounting for about one-quarter of the total population. Among them, acute myocardial infarction is the most dangerous risk factor for cardiovascular disease. There are about one million patients with acute myocardial infarction in China every year, and one in every three patients with myocardial infarction dies, with a mortality rate of more than 30% (Dong et al., 2023). The pathological cause of myocardial infarction is myocardial cell death due to long-term ischemia and is clinically defined as myocardial injury detected by abnormal cardiac biomarkers in the presence of evidence of myocardial ischemia. Studies have shown that during cardiac overload, different types of protein biomarkers associated with AMI disease are automatically released into the patient’s serum and plasma, such as myoglobin (Myo), cardiac troponins I and T (cTnI and cTnT), tumorigenicity suppressor 2 (ST2), brain natriuretic peptide (BNP), and creatine kinase-MB (CK-MB) (Parker, 2019). Myocardial injury is commonly defined by cardiac troponin in clinical practice. When the blood cTn level increases beyond the 99th percentile reference limit, myocardial injury is defined (Gao et al., 2015). Other biomarkers such as CK-MB and myoglobin have poor sensitivity and specificity. Cardiac troponins I and T, which are components of the contractile apparatus of cardiomyocytes and are almost exclusively expressed in the heart, are the preferred biomarkers for assessing myocardial injury. No significant in cardiac troponin I values has been reported after noncardiac tissue injury. Troponin T is more complex, with biochemical data showing that in some cases damage to skeletal muscle or other organs can also lead to an increase in troponin T (Huang et al., 2009).

Regular checkups can detect most of the risk factors, reduce the incidence of myocardial infarction, and increase the cure rate, especially for people with a high prevalence of myocardial infarction, such as those with a family history of genetic predisposition, a history of healing, and related cardiovascular system diseases. The identification of biomarkers is an effective method of diagnosing AMI, not only for detecting the onset and progression of the disease, but also for evaluating and tracking the therapeutic effects of drugs (Shade et al., 2021). Cardiac troponin I has become the preferred marker for the diagnosis of myocardial infarction due to its high sensitivity and specificity (Tucker et al., 1997). The normal value of cardiac troponin I is generally less than 0.04 ng/mL, and the cut-off value should be less than 1.5 ng/mL (Cao et al., 2024). The realization of instant sensitive and accurate detection is a challenge at present. Many attempts have been made to use different probes and optimize the chromatographic effect in the hope of achieving sensitive detection. Li et al. (2023) and Zhang et al. (2018). Multiple biocomponent detection using shell and core nanomaterials. Song et al. (2023) successfully synthesized ZIF-8 nanoprobe with pomegranate structure for detecting cardiac troponin I, and its effect was much better than that of the traditional Au-LFIA. Wang’s team (Wang et al., 2023) used the one-pot method to synthesize AuPt@Fe_x_O_y_ nanoparticles to realize the ultrasensitive detection of cardiac troponin I and assist the rapid diagnosis of myocardial infarction. The nanozymes synthesized in this study are Fe_3_O_4_ nanoparticles, which are referred to as nanozymes because of their peroxidase activity. Due to its unique enzymatic activity, it is widely used in lateral immunochromatography to achieve ultrasensitive detection of targets; Liu et al. (2011) used Fe_3_O_4_ nanoparticles as chromatographic chromogen to detect pesticide methyl paraoxon residues. Wang B. et al. (2022) used Fe_3_O_4_ colorimetric labeling and used the nanozyme effect to detect extracellular vesicles in plasma.

In this study, Fe_3_O_4_ enzyme nanoparticles (ENPs) with uniform size and high dispersion were successfully synthesized by hydrothermal method, with an average particle size of 200nm, which has special enzyme activity. The detection process is different from the traditional Au-LFIA, and it needs to use the chromogenic substrate TMB again after the first chromatography to enhance the effect. The purpose is to obtain more sensitive and accurate results. The detection limit of the cTnI E-LFIA was 1.5 ng/mL, while the sensitivity of the commercial cTnI Au-LFIA was 5 ng/mL. The sensitivity of the E-LFIA is about 3.3 times higher than that of the commercial cTnI Au-LFIA. It is the catalytic function of the ENPs that can amplify the signal.

We are committed to designing a rapid testing device that can be used in scenarios such as home self-testing, community broad-spectrum screening, hospital bedside rapid testing, and emergency ambulance diagnosis. Nanozymes, as a technologically mature material, possess excellent stability, which provides the basis for product conversion. In this study, materials science, immunology, biochemistry and other multidisciplinary knowledge were integrated, and magnetic and catalytic ferric oxide nanozymes were applied to the flow measurement immunochromatography platform to provide reference for interdisciplinary research. Previously, nanozymes have also been widely used in biosensing. Although its excellent magnetic properties are mostly used for enrichment of target materials, such application was not involved in this study. In addition, the wet sampling method used in this study was a common method in applied research, but there are some limitations in practical application and commercial promotion. At the same time, the detection limit of target cTnI in this study did not meet the clinical safety limit, which is also the direction of our subsequent optimization design.In summary, the LFIA designed here is fast, accurate, economical and easy to use without the aid of complex instruments. Nanozymes catalyze signal amplification and enhance color development to obtain higher sensitivity, which can be applied to the detection of a variety of molecules and disease diagnosis, with broad application prospects.

## Data Availability

The original contributions presented in the study are included in the article/Supplementary Material, further inquiries can be directed to the corresponding author.
