# Supplementary material for: A ultra-stable point of care nanozyme-based kit for cTnI detection in human serum
Source: Front Bioeng Biotechnol. 2025 May 15;13:1570668. doi: 10.3389/fbioe.2025.1570668 (PMC12119664; doi:10.3389/fbioe.2025.1570668)
Supplement: Supplementary file 1 [file DataSheet1.docx]

Supplementary Material

# Reagents and Materials

# Relevant biochemical reagents: Ethylene glycol, FeCl_3_-6H_2_O, NaAc, Ethanol, EDC, NHS, Tris buffer, Boric acid buffer were purchased from Shanghai Aladdin Biochemical Technology Co.,Ltd, and TMB Chromogen Solution was purchased from Beyotime Biotech Inc.

Mouse anti-cTnI mAb 1, Goat anti Mouse IgG was purchased from Shanghai Linc-Bio science Co.Ltd. Myo, CK-MB, H-FABP recombinant protein was purchased from Hangzhou Biogenome Biotechnology Co., Ltd. The materials required for LFIA, NC film, glass fiber, absorbent paper, and backing board, were purchased from Shanghai Jiening Biotech Co., Ltd.

The sample diluents were prepared according to our own scheme. 0.25%BSA and 0.1%Tween-20 were added into the PBS buffer, and the pH was adjusted to 7.0 by HCl. The raw materials were purchased from Aladdin Biochemical Technology Co., Ltd.

# Supplementary Figures and Tables

## Supplementary Figures


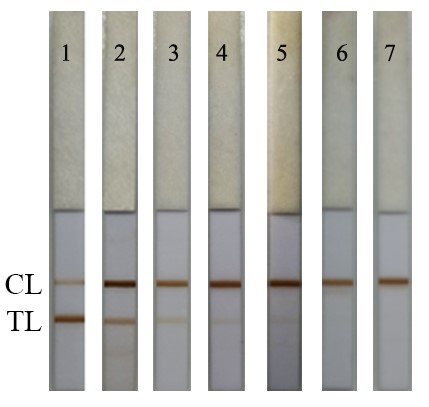


**Figure S1.** Sensitivity testing. Raw images of assay results for different concentrations of cTnI solutions. From 1 to 7, the concentrations are 500, 10, 5, 2, 1.5, 1 and 0 ng/mL.

**
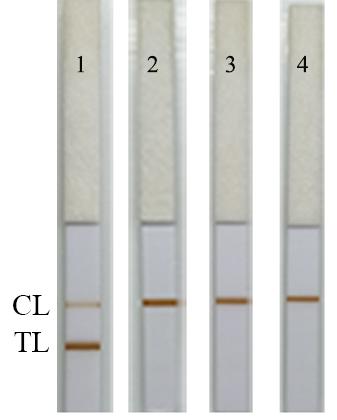
**

**Figure S2.** Raw images for specificity detection. From 1 to 4, cTnI, H-FABP, CK-MB, Myo.


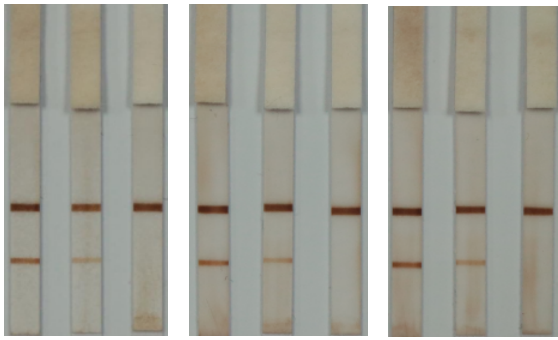


first batch second batch third batch


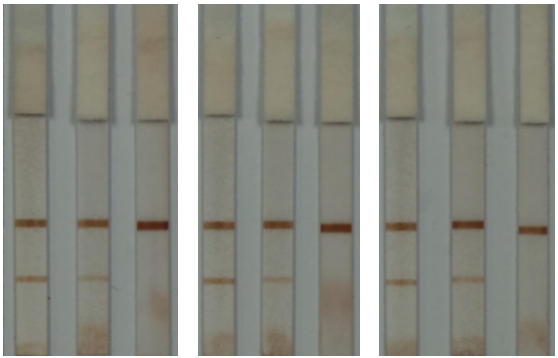


5day 10day 15day

**Figure S3.** Raw images of different batches (top) and different times of detection (bottom).

**
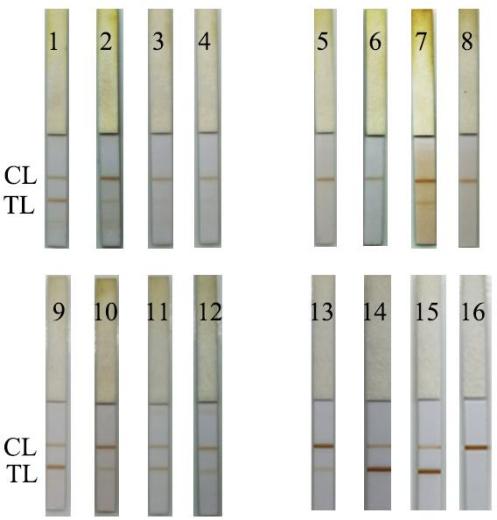
**


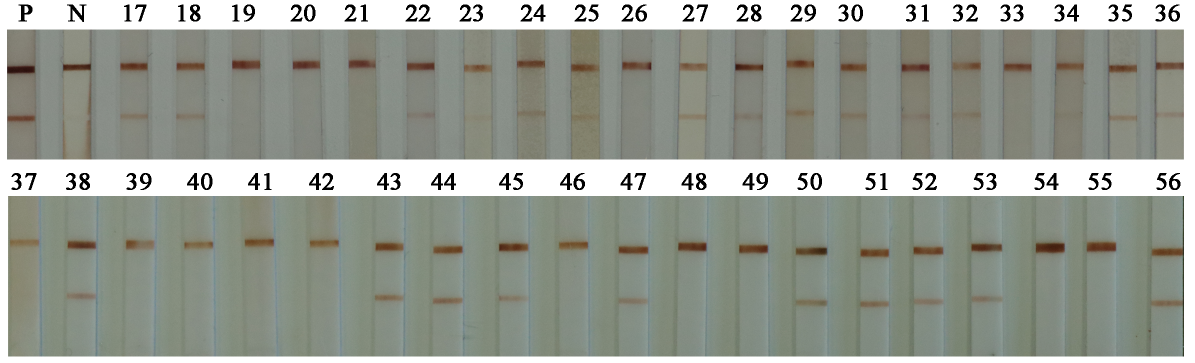


**Figure S4.** Raw images were detected in 76 clinical samples (another 20 negative samples were presented, and the sample information was shown in Table S2).

**
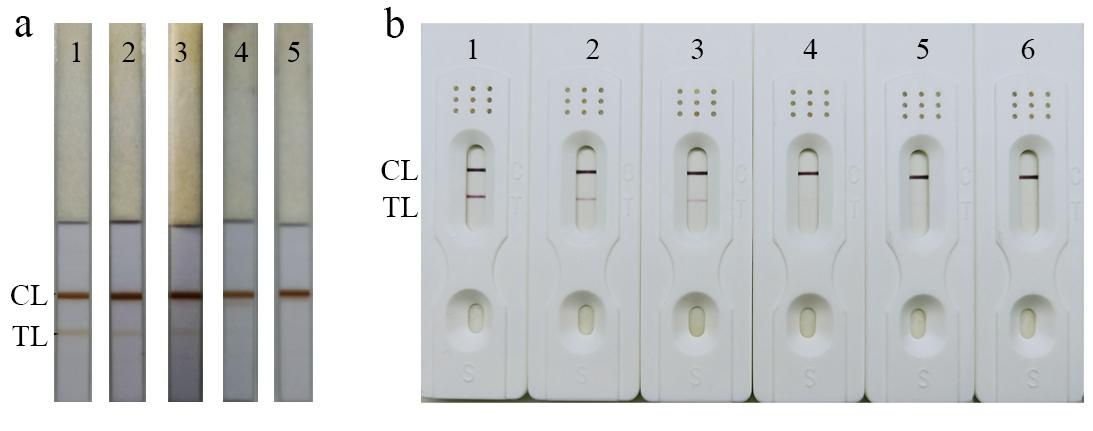
**

**Figure S5.** Left (a) shows the effect of MNPs-LFIA chromatography from 1 to 5 concentrations of 10, 5 , 1.5, 1 and 0 ng/mL. Right (b) shows the results of AuNPs-LFIA assay from 1 to 6 in the order of 50, 10, 5, 2, 1 and 0 ng/mL.

## Supplementary Tables

**Tables S1.** Basic information and detection results of the serum contributors. (The bold font indicates the positive samples that were not detected)

| No. | Gender | Age | ELISA result | LFIA intensity |
| --- | --- | --- | --- | --- |
| 1 | M | 35 | Positive | + + + |
| 2 | M | 64 | Positive | + + |
| 3 | F | 35 | Positive | + |
| **4** | **M** | **45** | **Positive** | **-** |
| **5** | **F** | **54** | **Positive** | **-** |
| 6 | F | 65 | Positive | + |
| 7 | F | 88 | Positive | + + + |
| **8** | **M** | **45** | **Positive** | **-** |
| 9 | F | 41 | Positive | + + + |
| 10 | M | 24 | Positive | + |
| 11 | M | 60 | Positive | + + |
| 12 | F | 55 | Positive | + |
| 13 | M | 58 | Positive | + |
| 14 | F | 49 | Positive | + + + + |
| 15 | M | 73 | Positive | + + + + |
| **16** | **M** | **56** | **Positive** | **-** |
| 17 | F | 36 | Positive | + + |
| 18 | M | 45 | Positive | + + |
| **19** | **M** | **63** | **Positive** | **-** |
| 20 | F | 39 | Negative | - |
| 21 | F | 72 | Negative | - |
| 22 | F | 52 | Positive | + + |
| 23 | M | 46 | Positive | + |
| 24 | F | 36 | Positive | ++ |
| 25 | F | 61 | Positive | + |
| 26 | M | 49 | Negative | - |
| 27 | M | 48 | Positive | + + |
| 28 | F | 39 | Positive | + + |
| 29 | M | 44 | Positive | + + + |
| 30 | M | 29 | Positive | + + |
| 31 | F | 36 | Positive | + + |
| 32 | F | 42 | Positive | + + |
| 33 | M | 60 | Negative | - |
| 34 | M | 48 | Positive | + |
| 35 | F | 43 | Positive | + + |
| 36 | M | 47 | Positive | + + |
| **37** | **F** | **56** | **Positive** | **-** |
| 38 | M | 55 | Positive | + + + |
| 39 | F | 47 | Negative | - |
| 40 | F | 61 | Negative | - |
| 41 | M | 48 | Negative | - |
| 42 | F | 52 | Negative | - |
| 43 | M | 44 | Positive | + + + |
| 44 | M | 53 | Positive | + + + |
| 45 | M | 42 | Positive | + + |
| 46 | F | 37 | Negative | - |
| 47 | M | 58 | Negative | + |
| 48 | F | 43 | Negative | - |
| 49 | F | 55 | Negative | - |
| 50 | M | 58 | Positive | + + + |
| 51 | F | 49 | Positive | + + + |
| 52 | F | 43 | Positive | + + |
| 53 | M | 45 | Positive | + + |
| 54 | F | 60 | Negative | - |
| 55 | M | 38 | Negative | - |
| 56 | F | 55 | Positive | + + + |

**Tables S23.** Basic information and detection results of the negative serum contributors. (Negative test results were not presented in the manuscript)

| 1 | M | 54 | Negative | - |
| --- | --- | --- | --- | --- |
| 2 | M | 49 | Negative | - |
| 3 | F | 56 | Negative | - |
| 4 | F | 34 | Negative | - |
| 5 | M | 61 | Negative | - |
| 6 | F | 59 | Negative | - |
| 7 | F | 71 | Negative | - |
| 8 | M | 65 | Negative | - |
| 9 | M | 29 | Negative | - |
| 10 | F | 54 | Negative | - |
| 11 | F | 67 | Negative | - |
| 12 | M | 38 | Negative | - |
| 13 | M | 45 | Negative | - |
| 14 | M | 55 | Negative | - |
| 15 | F | 65 | Negative | - |
| 16 | M | 39 | Negative | - |
| 17 | F | 55 | Negative | - |
| 18 | F | 42 | Negative | - |
| 19 | M | 61 | Negative | - |
| 20 | F | 48 | Negative | - |
